# Positive psychiatry in Ayurveda: A Historical review

**DOI:** 10.1192/j.eurpsy.2025.2020

**Published:** 2025-08-26

**Authors:** W. Upadhyaya, S. Bhat

**Affiliations:** 1 Thriftwood College; 2 Old age Psychiatry, Essex partnership University NHS Foundation Trust, Chelmsford, United Kingdom

## Abstract

**Introduction:**

Ayurveda is Indian traditional medicine that has a considerable presence in Europe. Incidentally the definition of health developed by the WHO since 1946 has striking similarities with the ones found in Ayurveda texts dating back few millenia ago. The encouragement Ayurveda provides in the pursuit of a flourishing life resonates with the principles and philosophy of Positive psychiatry. So this begs the question did Ayurveda have concepts resembling positive psychiatry and if so, what were the tenets. To this aim we review an Ayurveda text dating back to 3000 BC called Charaka Samhita.

**Objectives:**

To explore concepts related to positive psychiatry and psychology in Charaka Samhita.

**Methods:**

Relevant chapters and sections in Charaka Samhita were screened for descriptions or recommendations for mental health and a meaningful life.

**Results:**

Similarities between Positive Psychiatry and Ayurveda Psychiatry were present. As a part of psychotherapy Ayurveda recommends cultivation of spiritual awareness, wisdom fortitude/resileance and practice meditation. It further encourages the pursuit of ethically reasonable desires, material prosperity and righteous- religious conduct. For the healthy individuals, it recommends maintaining robust physical and mental health, actively accumulating wealth ethically and attain spiritual liberation.

**Conclusions:**

We conclude Ayurveda had its own version of Positive Psychiatry and delineates ways to achieve it.

**Disclosure of Interest:**

None Declared

